# Optimizing Deep Learning Model for Software Cost Estimation Using Hybrid Meta-Heuristic Algorithmic Approach

**DOI:** 10.1155/2022/3145956

**Published:** 2022-10-04

**Authors:** Ch Anwar ul Hassan, Muhammad Sufyan Khan, Rizwana Irfan, Jawaid Iqbal, Saddam Hussain, Syed Sajid Ullah, Roobaea Alroobaea, Fazlullah Umar

**Affiliations:** ^1^Department of Computer Science, Capital University of Science and Technology, Islamabad 44000, Pakistan; ^2^Department of Creative Technologies, Air University, Islamabad 44000, Pakistan; ^3^Department of Computer Science, University of Jeddah, Jeddah, Saudi Arabia; ^4^School of Digital Science, Universiti Brunei Darussalam, Jalan Tungku Link, Gadong, BE1410, Brunei Darussalam; ^5^Department of Electrical and Computer Engineering, Villanova University, Villanova, PA, USA; ^6^Department of Computer Science, College of Computers and Information Technology, Taif University, P.O. Box 11099, Taif 21944, Saudi Arabia; ^7^Department, Khana-e-Noor University, Pol-e-Mahmood Khan, Shashdarak, 1001 Kabul, Afghanistan

## Abstract

Effective software cost estimation significantly contributes to decision-making. The rising trend of using nature-inspired meta-heuristic algorithms has been seen in software cost estimation problems. The constructive cost model (COCOMO) method is a well-known regression-based algorithmic technique for estimating software costs. The limitation of the COCOMO models is that the values of these coefficients are constant for similar kinds of projects whereas, in reality, these parameters vary from one organization to another organization. Therefore, for accurate estimation, it is necessary to fine-tune the coefficients. The research community is now examining deep learning (DL) as a forward-looking solution to improve cost estimation. Although deep learning architectures provide some improvements over existing flat technologies, they also have some shortcomings, such as large training delays, over-fitting, and under-fitting. Deep learning models usually require fine-tuning to a large number of parameters. The meta-heuristic algorithm supports finding a good optimal solution at a reasonable computational cost. Additionally, heuristic approaches allow for the location of an optimum solution. So, it can be used with deep neural networks to minimize training delays. The hybrid of ant colony optimization with BAT (HACO-BA) algorithm is a hybrid optimization technique that combines the most common global optimum search technique for ant colonies (ACO) in association with one of the newest search techniques called the BAT algorithm (BA). This technology supports the solution of multivariable problems and has been applied to the optimization of a large number of engineering problems. This work will perform a two-fold assessment of algorithms: (i) comparing the efficacy of ACO, BA, and HACO-BA in optimizing COCOMO II coefficients; and (ii) using HACO-BA algorithms to optimize and improve the deep learning training process. The experimental results show that the hybrid HACO-BA performs better as compared to ACO and BA for tuning COCOMO II. HACO-BA also performs better in the optimization of DNN in terms of execution time and accuracy. The process is executed upto 100 epochs, and the accuracy achieved by the proposed DNN approach is almost 98% while NN achieved accuracy of up to 85% on the same datasets.

## 1. Introduction

Software project development includes different sets of activities, from requirements collection to testing and maintenance, which need to be executed within a specified time period and budget to achieve a reliable software product [1].

Because of the high rate of change in customer needs and rapid technological advancement, software development is more complicated than other types of engineering projects. This makes it difficult for effective software project management to achieve specific goals while adhering to a set of constraints [2]. The Standish Group report shows that only 32% of the software projects are successfully delivered on time, within the allocated budget, and have the required functionality. 44.4% did not fulfil the aforementioned requirements, and 24.4% failed; that is, they were either cancelled or completed but never used [3]. Another survey study, which was conducted among 800 senior IT managers in the US, Germany, Singapore, UK, Japan, and France, shows the same results: 62% of projects were not completed on time, 49% went over budget, and 47% required extensive maintenance [4].

Project planning is the most critical aspect of software project management and consists of a set of managerial and technical practices that can be broadly classified as the development of the project plan, execution of the project plan, and anticipating problems that may arise and preparing tentative solutions to those problems. Software cost and effort estimation come under the project-planning phase and include the process of determining how much a project will cost, how many man-hours will be required to complete the project, and how long it will take. Inaccurate estimation can result in project failure and escalation in project costs. Some of the reasons for inaccurate estimation of software projects include the following: inaccurate project goal setting, project scheduling, required development effort (capability, estimation, and availability), project budgeting, project risk management, stakeholder politics, and market pressures [5]. Effective estimates are critical in the decision-making process. Estimation must take into account both the market and the organisational perspective in order to control project costs and scope and manage the project in accordance with organisational policies. Project effort underestimation may end up in a situation where, because of a shortage of budget and time commitments, work cannot be accomplished whereas overestimation may end up with the rejection of a project proposal [6].

Several methods for solving the estimation problem are presented in the existing research literature. This effort can be divided roughly into two categories: there are two types of methods: algorithmic methods and nonalgorithmic methods [7]. Nonalgorithmic approaches rely on deduction and analogy in their estimation processes. For estimation, these models require knowledge of previously completed projects that are similar to current software projects. Previous software projects or dataset analyses are used to make estimates. Estimation techniques based on nonalgorithmic models [8] include analogy estimation, expert judgement techniques (including top-down and bottom-up estimation), learning-based methods (artificial neural network (ANN) [9–11], machine learning (ML) [12], and case-based reasoning (CBR) [13]), regression, and fuzzy logic [14].”

In the case of fuzzy logic-based methods [15, 16], although no training is required, for complex features, cost estimation becomes tedious. Furthermore, fuzzy models are difficult to use. In the case of expertise-based methods, experts determine possible costs and factors impacting the estimate. Therefore, the estimation accuracy is based on expert skill, knowledge, and experience. Learning-based techniques automatically identify trends and patterns. It gains experience and keeps improving in efficiency and accuracy. Multidimensional data of various varieties can be handled easily through learning-based algorithms. There are also some limitations, as it requires huge data sets to train on, which should be unbiased and inclusive. Also, it takes a considerable amount of time to train and learn to fulfil a considerable amount of relevancy and accuracy. The progress of the software development process has continued to evolve over the past few decades. Therefore, most of the available data sets are heterogeneous because their sources come from various organisational projects. On the other hand, there are a large number of missing values. Therefore, the use of neural networks for high-dimensional and multiobjective data classification is a challenging task.

In the algorithmic model [17], the cost estimation is provided by a mathematical model that utilises the attributes of products, projects, and processes. These equations are derived from research and involve parameters such as function points, source lines of code (SLOC), and cost drivers (such as design methodology, risk assessments, and language dependency). Some examples of algorithmic models are the SAIC model, function point-based models, Putnam's model, COCOMO, and SEERSEM model. COCOMO is the most extensively used regression algorithmic model for software cost estimation [18] because it allows users to adjust parameters according to the uniqueness of their projects. For estimation, COCOMO uses equations and parameters based on experience from earlier software projects. COCOMO II is an enhancement of COCOMO and is widely adopted because of its simplicity and accuracy. For effort estimation, it uses the project size (in terms of Kilo Source Line of Codes (KSLOC)) and 22 cost drivers, in which 05 scale factors and 17 effort multipliers are included. The outcome of the COCOMO II (postarchitecture model) is in terms of people per month for a project. However, a prominent limitation of algorithmic models is that they are difficult to learn and require data about the current state of the project. So basically, no one method can be regarded as the best method. Therefore, it is usually recommended to combine these methods (hybrid methods) to obtain better cost estimates.

COCOMO is a parametric effort estimation model. COCOMO model coefficients play a significant role in effort estimation. The limitation of the COCOMO models is that the values of these parameter coefficients are constant for similar kinds of projects whereas, in reality, these parameters vary from one organization to another organization. Therefore, it is difficult to have a single, acceptable, and logical parametric model. The software project dataset contains data on heterogeneous projects (different project indicators in terms of scale and attributes). Therefore, in order to estimate the accuracy, the parameters need to be fine-tuned [19]. In order to overcome the limitations of COCOMO, many studies have adopted different methods to adjust the coefficients of COCOMO II and improve cost estimation. In the estimation problem, an upward trend has been seen in the use of meta-heuristic algorithms inspired by nature. Because of their unique characteristics, such as large search spaces and random selection techniques, these meta-heuristic algorithms perform well in dealing with optimization problems in various fields of interest [20].

**Figure 1:**
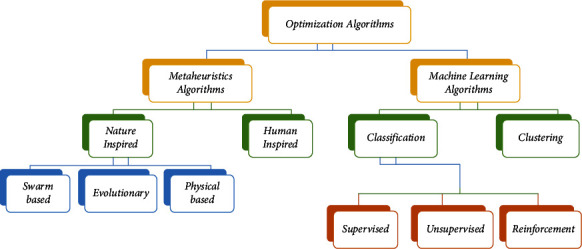
Optimization algorithms.

Deep learning (DL) is now being considered by the research community as a viable solution for improving cost estimation. Deep learning (DL) characteristics such as better feature selection and representation enable it to outperform other shallow learning techniques. Because DL makes it possible to express complex relationships between effort and cost drivers, it is a better choice for estimating software costs [21]. Although deep learning architectures offer some advantages over existing shallow technologies, they also have some drawbacks, such as long training times, overfitting, and underfitting. Meta-heuristic algorithms allow formulating the DL components into an optimization problem. The hybrid of ant colony optimization with the BAT (HACO-BA) algorithm is a hybrid optimization technique that combines the most common global optimum search technique for ant colonies (ACO) in association with one of the newest search techniques called the BAT algorithm (BA). The inclusion criteria of these algorithms are high maturity, state-of-the-art, and representative. Meta-heuristics, nature-inspired, and machine learning optimization algorithms are shown in Figure 1.

### 1.1. Contribution

RQ1: Which of the meta-heuristic algorithms has the lowest MRE, MMRE, MBRE, and PRED?

Ans: Various meta-heuristic algorithms are used for model optimization. From the literature review, we have to select different algorithms on the basis of their performance for solving various software estimation problems. Different parameters will be evaluated, for example, MRE, MMRE, MBRE, and PRED, to find out which performs better among the selected algorithms. Those algorithms are the best that have optimised values for evaluation parameters.

RQ2: whether the performance of the meta-heuristic algorithm changes or varies by changing the dataset.

Ans: The three most widely used datasets for software cost and effort estimation, that is, NASA dataset, COCOMO 81 dataset, and KEMERER dataset, will be used as input to the various models built using the mete-heuristic algorithm. The performance of all these models will be tested and evaluated on these three datasets to see whether their performance changes or varies by changing the datasets.

RQ3: nature-inspired and meta-heuristic algorithms combined with deep learning can improve the software estimation process.

The algorithm that performs best among the other selected algorithms in terms of improvement of the evaluation parameter from RQ2 is used to tune the deep neural network (DNN). The proposed model, which is a combination of meta-heuristics and DNN, is tested and evaluated for fast and efficient results.

RQ4: whether the performance of a meta-heuristic-based deep learning algorithm changes by changing the dataset.

Different datasets are chosen from the state of the art. These datasets are used as input to the proposed meta-heuristic-based DNN. Performance is tested and evaluated for this model by changing the datasets.

Whether metaheuristic-based deep learning algorithms perform better than the NN-based approach for software estimation.

Ans: Neural network (NN)-based approaches for software estimation are widely used by the research community. However, there are various pros and cons to using NN techniques. Solving various optimization problems with DNN is the most popular topic among researchers. So we have to check whether the meta-heuristic with DNN performs best when compared to the NN model.  Table 1 describes the notation guide for each algorithm as well as additional abbreviations.

The rest of the article is formatted as Section 2 demonstrates related work and a subsection of research gaps, Section 3 contains a problem statement, Section 4 describes the proposed methodology, and results are discussed in Section 5.

## 2. Related Work

Many researchers and practitioners around the world use different methods to improve software estimation [17]. The existing literature in the field of software estimation has proposed various techniques to evaluate the accuracy of predictive models. The most popular among the existing literature is the mean magnitude of relative error (MMRE), based upon mean relative error as shown in the following equation:

(1) $$\begin{array}{c} \text{MRE}=\frac{\text{actualefforts}-\text{predictedefforts}}{\text{actualefforts}}. \end{array}$$

The lower the MMRE value, the closer the predicted estimated value is to the actual estimate, and vice versa. There is work being done to review existing studies on software cost estimation [19]. Various meta-heuristic techniques for software cost estimation have been implemented over the last decade. There is work that computes the effectiveness of meta heuristics algorithms [17, 20, 22–24] related to the optimization of software cost estimation. For example, in the existing literature, genetic algorithm (GA) [23], hybrid GA [24], ants colony optimization (ACO) [25] algorithm, and firefly algorithm (FA) [26] improved cost estimation. Moreover, existing literature also demonstrates the effectiveness of meta-heuristic algorithms in terms of optimizing the parameters of COCOMO [25–29]. There is work to optimize COCOMO II coefficients using hybrid algorithms. A hybrid method is [30–36] the combination of several methods that can be derived from algorithmic or nonalgorithmic techniques. For example, the author of [34] uses the artificial bee colony and the genetic algorithm for optimization.

Bee colony optimization (BCO) [22] is a subclass of swarm intelligence that has been effectively used in a variety of engineering applications, including software estimation. By using BCO, COCOMO parameters have been optimised. In the proposed technique, artificial agents are produced by analogy with bees. Better results are obtained as compared to other models such as the Baily-Basil and Halsted models. Puri and Kaur [20] discussed various meta-heuristic techniques that are used for software cost and effort estimation. BCO works on the natural phenomenon of getting food from bees. It has two stages: moving forward and moving backward. FA is based on firefly flashing characteristics, and human opinion dynamics (HOD) is based upon the human creative problem-solving process to solve complex optimization problems.

The parameters of the basic COCOMO model are optimised using a simplified GA technique in [23]. We use the NASA software project dataset as a starting point. According to empirical evidence, the basic COCOMO model produces a superior actual estimate. The authors of [24] proposed the whale–crow optimization (WCO) algorithm, which is a combination of the whale (WOA) and crow search (CSA) optimization algorithms. The main objective of the WCO approach is to find an optimal regression coefficient to build an optimal regression model. The performance evaluation of the proposed scheme is calculated using four datasets of software estimation. MMRE is analysed, and a reduction is seen, which proves that WCO performs well in both the linear regression model and the kernel regression model. The authors of [26] proposed the firefly algorithm (FA) as a meta-heuristic optimization technique for optimizing three COCOMO-based model parameters. Among these three models, the basic COCOMO model and the other two models are proposed in the state-of-the-art as an extension of the basic COCOMO model.

Jafari and Ziaaddini [27] analysed the effectiveness of the harmony search algorithm (HSA). Using the NASA dataset, the work demonstrates a significant reduction of MMRE as compared to basic COCOMO. To estimate software reliability issues, the partial swarm intelligence (PSO) technique and ant colony optimization (ACO) are used [28]. In [29], the BAT algorithm is presented to improve the software estimation accuracy. The effectiveness of the BAT algorithm is compared with grey relational analysis (GRA). GRA delivers an effective solution for the complex interrelationships between multiple response parameters. The results show that GRA performs better in terms of error rate.

A new hybrid model, BATGSA [30], which is based on two meta-heuristic algorithms, gravitational search, and the BAT Algorithm, is proposed to achieve better software estimation. The BAT algorithm uses the random walk of BATs to determine the hunting and routing behaviour of bats in the exploration phase and uses the gravitational effect of GSA to further improve and speed up the search speed of BATs. Four NASA datasets are used for analysis, downloaded from the promise repository. The minimization of errors is obtained by comparing the COCOMO against the hybrid BATGSA algorithm.

The work presented in [31] demonstrates that software cost estimation can be improved by using a hybrid approach based on Tabu search and genetic algorithms. Furthermore, the hybrid model is used to tune the COCOMO.

In [32], three meta-heuristic optimization algorithms are implied synthetically in order to refine the COCOMO model: partial swarm optimization (PSO), invasive weed optimization (WOA), and genetic algorithm (GA). The dataset is divided into two groups: train and test datasets. Furthermore, the dataset is further divided based on the type of projects. COCOMO parameters are tuned using meta-heuristic algorithms, and the new cost drivers are tested using a testing dataset.

In [33], a hybrid approach based on ant colony (ACO) and chaos optimization algorithm (COA) is presented. In the first phase, the dataset is classified into two parts according to project type; one is used for training the model, and the second is used to test that trained model. Each algorithm (ACO, hybrid ACO-COA) is applied with its own function in the first phase. Next, the most optimal solutions obtained for estimation from the proposed model are then tested using testing data. In the result section, a comparison is made between COCOMO, ACO, and the hybrid ACO-COA model.

In [34], the author proposed a hybrid model based on genetic algorithm (GA) and artificial bee colony (ABC) schemes for optimization of estimation method parameters according to project size. In the artificial bee colony (ABC) algorithm, each bee presents a solution to the problem. In every iteration, the most graceful bee solution is chosen, and their fitness is calculated. The bees whose performance is low are replaced with new bees. The new bee population is generated using GA crossover, selection, and mutation. Finally, the most optimal solution is found. The results demonstrate that GA, ABC, and the hybrid model of ABC with GA performed well (fewer MRE error values) compared to the COCOMO model. Moreover, the hybrid model demonstrates better convergence as compared to the GA and ABC algorithms.

In [35], the author uses a hybrid model formed on PSO and DE algorithms to deliver a more comprehensive and efficient estimate. With incomplete and ambiguous input data, the hybrid model works well, and it can operate reliably in software estimation. In the proposed hybrid model, the accuracy of PRED (25) increased 1.34 times.

In [36], a hybrid model based on the cuckoo search (CS) and harmony search (HS) algorithms is used for optimizing COCOMO-II coefficients. The proposed CSHS has two stages. CS at the first stage is used for finding the initial optimal solution for local and global search. In the second, a new harmony vector or solution is generated using the HS strategy, which is compared with the global best solution found in the first stage. If it has better fitness, then the global best is replaced. This procedure is continued until the given iterations are completed. The proposed technique is applied to NASA 93 datasets. The aim is to achieve an estimation value close to the actual value.

ML is a method that trains computing systems to improve itself by learning from previous data available. ML programs work by constructing a prediction model from a set of previously available training data, and this step is followed by the data-driven predictions [37].

Categorically, analysis was performed in [21] on 4 neural network models: (1) general regression NN (GRNN), (2) multilayer perceptron (MLP), (3) cascade correlation neural network (CCNN), (4) radial basis function NN (RBFNN), and mean absolute residual (MAR) were the criteria used for performance evaluation. Four inputs have been assigned to each model, including (1) development platform, (2) software size, (3) resource level, and (4) language type. Five datasets mined from the ISBSG are used. The output of the model was software effort.

In [38], an empirical study was conducted by using the random forest (RF) method for software effort estimation. In RF, first, we investigate the number of trees affected and then evaluate the number of selected attributes for growing trees. The outcomes show that the estimation accuracy is very sensitive to these parameters.

In addition, the survey conceded that we optimize the RF model by selecting the best values for these two parameters. We compare the performance of the enhanced RF model with the performance of the classical regression tree (RT) by using the (70–30) hold-out validation method and using three COCOMO, IBSSG, and Tukutuku data sets.”

Some highly mature and popular ML algorithms, such as support vector machines, regression, decision trees, RF, Bayesian inference, ANN [39], and feature selection, are an important process during training a model because model efficiency depends on selected variables. It is very important to choose features that have a significant influence on the prediction model [40].

Barmpalexis et al. [41] accelerate the neural network training process by using optimized particle swarm optimization (OPSO). The main function of OPSO is to enhance the free PSO parameters by having a new swarm within a swarm. The aim is to build a quantitative model by applying the OPSO technique to neural network training. This method yields the parameter combinations needed to improve the overall performance of the optimization process.

In [42], the author proposed a nonalgorithmic method for estimating software development effort. This article discusses the integration of wavelet neural networks (WNN) and meta-heuristic methods for estimating software development effort (SDEE). The technologies used here are WNN with the firefly algorithm and the BAT algorithm. As the activation function in WNN, two wavelet function variants—Morlet and Gaussian—are used. It has been discovered that using WNN with the firefly and BAT algorithms (FA and BA) produces better results than using simple WNN without any meta-heuristics. According to the author's experimental results, the WM technique performs the worst across all four data sets. However, combining meta-heuristics with WNN yields significantly better results.

In [43], the author conducted an exploratory longitudinal case study. Data collection was conducted through semistructured interviews and archival research. The two-stage estimation process, which reestimates in the analysis stage, improves the effort estimation accuracy.

Underestimation is the main trend in software evaluation, and less mature teams experience greater work overspending.

Some of the most common challenges are solved in large-scale agile software development. In order to improve the effort estimation, the team maturity, distribution, and demand size and priority need to be considered.

Emary et al. [44] proposed a modified grey wolf optimization (GWO) that utilises the reinforcement learning rules or principles by integrating them with neural networks in order to improve the model performance. The combination of GWO with a neural network forms experienced GWO (EGWO). The performance of experienced GWO is measured by finding the optimal weights of the neural network and choosing a subset of related features (predictors, variables) for use in model building.

The author in [45] presents a comprehensive dataset for the story points-based estimation. 23,313 issues from 16 open-source projects are addressed. A prediction model using DNN is also proposed for the estimation of the story points.

In [46], the author proposed a new technique of deep learning modified neural network using the cuckoo search algorithm for initialising the weight of the network and applying HPSO to obtain a better classification of various parameters of the dataset. A neural network is qualified as a “deep network” when there are more than three layers. The deep modified neural network (MNN) classifier is comprised of neurons with weights in addition to biases. Also, the deep MNN classifier consists of different sorts of layers: convolution, pooling, as well as a fully connected layer. The cuckoo search algorithm (CSA) is used to initialise the weight of the network. The input of this deep MNN is the effort multiplier, that is, the software development of the COCOMO dataset, database size, exponent value, constant value, etc. This step is carried out through an optimization process, which is executed by using hybrid particle swarm optimization (HPSO) with genetic operators. (Crossover, explicitly, and also mutation of genetic algorithms). While choosing the NN weights, which help to enhance the classification of the model, HPSO is used to attain better classification outcomes. Finally, the proposed deep MNN is evaluated on different performance parameters: relative error (RE), magnitude of relative error (MRE), mean-MRE (MMRE), mean balanced relative error (MBRE), and also percentage of prediction (PRED) and compared with traditional NN. The experiment shows better results in all features. The execution time for effort estimation is also compared. When 10 instances are considered, the proposed deep MNN shows a decrease in execution time compared with the traditional NN. On the other hand, there is also a limitation in the proposed technique; that is, when the instances were increased to 50, the results show that the proposed method takes more time.

A hybrid approach is introduced in [11], which is divided into two sections. First, the author applies the GA for feature optimization. To obtain the desired results, the total population is divided into several subpopulations and applies computation for each population, which includes designing of chromosomes and calculation of fitness functions. In the second section, an improved DNN for classification is proposed. As we know, neural networks show significant performance in terms of classification. However, neural networks cannot classify multiobjective functions or high-dimensional data, so the paper proposes an enhanced DNN model with sparse auto-encoders to overcome this limitation. We use the proposed technique to learn the feature pattern. The adaptive auto-encoders are used in conjunction with the denoising model to produce better results for specific software features. The MATLAB tool is used for experiments in various scenarios that are performed for software defect prediction. The proposed technique's performance is assessed using data sets KC1 and CM1. A comparative study reveals that the proposed technique outperforms the experimental scenario without optimization in all four scenarios created during the experiments. A brief summary of estimation methods and their limitations is shown in Table 2.

For categorised problems, the deep belief network (DBN) is an excellent machine learning technology. The traditional DBN, on the other hand, does not function well for unbalanced data classification because it assumes that each class has the same cost. Cost-sensitive approaches are employed to overcome this issue, which attaches varying misclassification costs to different classes without affecting the actual data sample distribution. The author [47] proposes an evolutionary cost-sensitive deep belief network (ECS-DBN) model in which he first optimises the misclassification costs using optimization algorithms that automatically update their corresponding parameters and then applies them to the deep belief network. The author demonstrated that ECS-DBN outperforms other competing techniques significantly. The suggested ECS-DBN improves DBN by applying cost-sensitive learning techniques. The adaptive differential evolution approach is utilised in practise to find the misclassification cost and solve the unknown misclassification cost [48–50]. In [51], the grey wolf algorithm (GWO), the strawberry algorithm (SBA), and the harmony search algorithm (HSA) were tested on MRE and MMRE parameters using the NASA dataset.

The author proposed multiple approaches to make the software secure and reduce future efforts for maintance. In [52], the author proposed a lightweight identity-based signature scheme for content poisoning mitigation in named data networking with the Internet of things. A secure identity-based generalised proxy signcryption (IBGPS) scheme that is lightweight and provable for the industrial Internet of Things (IIoT) is proposed in [53]. For the Internet of Things-enabled smart grid, CBSRE is a lightweight and formally secure certificate-based signcryption with proxy reencryption. For the named data networking-enabled Internet of things, a lightweight heterogeneous generalised signcryption (hgsc) scheme, securing the NDN-based Internet of Health Things with a low-cost encryption scheme, is proposed in [54, 55]. Machine and deep learning approaches are widely used in different areas of life. The author proposed an intrusion detection system for IoT based on deep learning and a modified reptile search algorithm in [56] and a modified Aquila optimizer for forecasting oil production in [57]. In [58], author forecasted the wind power using the marine predator algorithm and mutation operators for wind power forecasting to evaluate the performance of meta heuristic approach.

## 3. Algorithmic Approaches

Meta-heuristic algorithms and nature-inspired algorithmic approaches are discussed in this section.

### 3.1. Meta-Heuristic Algorithms

Nature influenced meta-heuristic algorithms and nature-inspired algorithms. Natural biological systems, evolution, human activities, animal group behaviours, and other factors can inspire algorithms, such as the biological human brain-inspired artificial neural network [1], the genetic algorithm stimulated by evolutionary theory [2], and Dujuan. The cuckoo search algorithm (CSA) was inspired by the cuckoo's birth behaviour [3], whereas the grey wolf optimization (GWO) was inspired by the grey's aggressive behaviour [4].

It is found that these nature-inspired algorithms are more effective and efficient than traditional algorithms in solving real-world optimization problems because they can effectively deal with highly complex and nonlinear problems, especially in the fields of science and engineering [22]. Meta-heuristic is defined as an iterative method, which explores and uses search space to guide lower-level heuristics by intelligently combining different concepts. They are inspired by observing phenomena that occur in nature. The summary of the used meta heuristics techniques is presented in Table 3.

#### 3.1.1. Ant Colony Optimization

**Figure 2:**
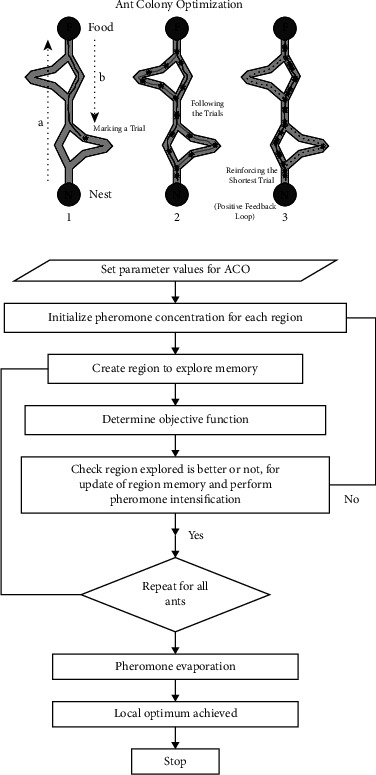
Ant colony algorithm.

Marco Dorigo was first introduced in 1992 as a multiagent solution for optimization problems. When an ant moves, it deposits pheromone (in varying amounts) on the ground and uses the smell of this substance to determine its path. The colony's other members follow the path to find food and return to the nest in the same manner. Ants begin their search for food sources by randomly exploring the area around their nest [25]. When ants find a food source, they evaluate the quality of the food and bring a small amount back to their nest. The flow chart of the ant colony optimization algorithm is shown in Figure 2.

(2) $$\begin{array}{c} \tau_{i,j}\left(t+1\right)=\tau_{i,j}\left(t\right)+\sum_{k=1}^{n_{k}}\delta \tau_{i,j}^{k}\left(t\right). \end{array}$$

Ants communicate via indirect channels and coordinate their activities in a hidden state by doing changes in the surrounding environment as shown in equation (2). ACO works on the principle of indirect artificial communication to match societies of artificial agents. The steps of ant colony optimization algorithms are shown in Algorithm 1.

#### 3.1.2. BAT Algorithm

**Figure 3:**
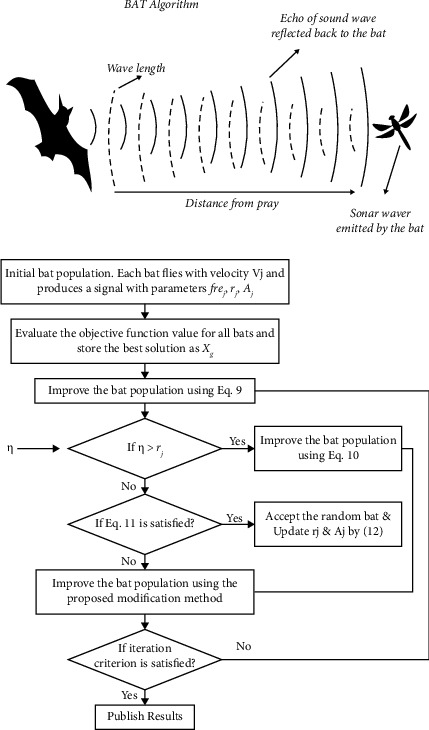
BAT algorithm.

The BAT algorithm (BA) [30, 42] was proposed by Xin-She Yang and is influenced by mini-BAT echolocation behaviour. BATs use this behaviour to direct and assist them in flight and hunting. BATs can not only move but also discern between obstructions and bug forms, even in complete darkness, thanks to their incredible orientation mechanism. The flow chart of the BAT algorithm is shown in Figure 3.

The position of each BAT in the search space is defined by *x*_*k*_^*t*^, frequency *f*, velocity *v*_*k*_^*t*^, loudness *A*_*k*_^*t*^, and transmitted pulse rate *r*_*k*_^*t*^ in this algorithm. The velocity and position *k*^*th*^ of the BAT at time *t* are calculated using the Equations 3rd, 4th, and 5th.

(3) $$\begin{array}{c} f_{k}=f_{\min}+\left(f_{\max}-f_{\min}\right)\beta ,\\ v_{k}^{t}=v_{k}^{t-1}+\left(x_{k}^{t-1}-x_{k}^{t}\right)f_{k},\\ x_{k}^{t}=x_{k}^{t-1}+v_{k}^{t}. \end{array}$$

Among them, *f*_*k*_ is the frequency of the sound waves emitted by *k*^*th*^ BATs; *f*_*mix*_ and *f*_*man*_ are the minimum and maximum sound waves frequencies, respectively; *β* is composed of uniform which distributes the random number generated by [0, 1]. The velocities of *k*^*th*^ BAT are *v*_*k*_^*t*^ and *v*_*k*_^*t*−1^ at time *t* and time (*t* − 1), and *x*_*k*_^*t*^ represents the current global optimal position of the BAT.

For local search, each bat the position is measured using equation (6). Each BAT local random walk is calculated by using the following equation:

(4) $$\begin{array}{c} X_{\text{new}}=X_{\text{old}}+\delta \text{Ĺ}\left(t\right), \end{array}$$

where *δ* is a random number produced uniformly distributed on the interval [−1, 1], *X*_*old*_ is a solution arbitrarily selected from the current optimal solution, in the iteration of *i*^*th*^, Ĺ is the normal uproar of all BATs. The steps of the BAT algorithm are shown in Algorithm 2.

#### 3.1.3. Hybrid of Ant Colony Optimization with BAT (HACO-BA)

The hybrid of ant colony optimization with BAT (HACO-BA) algorithm is a hybrid optimization technique that combines the most common global optimum search technique for ant colonies (ACO) in association with one of the newest search techniques called the BAT algorithm 3 (BA).

### 3.2. Performance Analysis and Evaluation

In this section, we discuss about the performance analysis, experimental setup, datasets selection, evaluation metrics, and experimental results.

#### 3.2.1. Performance Analysis

In the experimental section, we will compare the performance of 6 meta-heuristic algorithms, that is, GWO, GA, strawberry (SBA), cuckoo search, particle swarm (PSO), and ant colony optimization (ACO) [23, 25, 28, 34, 35, 59]) that use meta-heuristic algorithms in terms of effort and cost estimation with each other and with COCOMO model.

In 1981, Barry Boehm proposed the constructive cost model (COCOMO). It is the most often used algorithmic or parametric model. The model parameters and equations are generated from historical projects for estimation. The model is used to estimate the project's size, the amount of effort necessary, and the project's cost. We must first construct a set of criteria to quantify this. In this model, we use functional points (FP) and lines of code (LOC) to compute the required efforts. The “person month” unit is used to estimate effort in this method, which is equivalent to a single person's month efforts. COCOMO models are divided into two categories.

- COCOMO I
- COCOMO II

COCOMO I (also known as COCOMO 81) and COCOMO II (COCOMO 2000) were released in 1981 and 1995, respectively. It was, however, published in the year 2000. COCOMO I is separated into three levels of difficulty: basic, intermediate, and advanced [1]. The formulas utilised to calculate the estimations change across these methods. The most widely utilised methods are basic and intermediate; these approaches are further divided into three sections or measurements. These are organic, semidetached, and embedded, according to the projects featured in it [2].

Basic COCOMO *E*, organic COCOMO *E*_*B*−*O*_, semidetached COCOMO *E*_*B*−*S*_, and embedded COCOMO *E*_*B*−*E*_ formulas are shown in the following equations, respectively.

(5) $$\begin{array}{c} E=a{\left(\text{size}\right)}^{b}, \end{array}$$

(6) $$\begin{array}{c} E_{B-O}=2.4*{\left(LOC\right)}^{1.05}, \end{array}$$

(7) $$\begin{array}{c} E_{B-S}=3.0*{\left(LOC\right)}^{1.12}, \end{array}$$

(8) $$\begin{array}{c} E_{B-E}=3.6*{\left(LOC\right)}^{1.20}. \end{array}$$

These are the fundamental COCOMO equations. KLOC represents the number of code lines and project size in these equations. The COCOMO coefficients “*a*” and “*b*,” as well as the value of “*E*,” describe the required efforts.

#### 3.2.2. Experimental Setup

This stage is building a model in MATLAB software to estimate effort using six algorithms selected for their excellent performance in diverse optimization situations [25–27]. Because its basic data element is a matrix, and its capability can be easily increased by utilising multiple toolboxes, using MATLAB software provides significant advantages. On three publicly available datasets retrieved from the promise repository, various tests are carried out using cutting-edge algorithms.

#### 3.2.3. Datasets Selection

Effort multipliers are taken as input to the models, from the following defined datasets. These effort multipliers are categorized into three groups, which are as follows:

- Positively correlated to additional effort
- Negatively correlated to additional effort
- Containing just schedule information

*(i) NASA*. The NASA dataset was obtained from the promise software engineering repository, which can be used publicly to improve software cost estimation methods. It includes 93 software project information, which has been recorded for many years from several NASA centers. Dataset contains 15 effort multipliers and 5 scaling factors, which have different values in each software project.

*(ii) COCOMO 81*. This dataset is also known as COCOMO 81 which is publicly available on promise software engineering repository. This repository's software project data are stored in the COCOMO software cost model, which calculates the amount of effort required to develop software projects in a calendar month. It also includes a standard effort multiplier.

*(iii) KEMERER*. KEMMER dataset is measured in KLOC. It is used in many machine learning applications for software engineering which has 8 attributes. To compare it with Nasa and COCOMO dataset, which has 15 attributes, we assume the rest of attribute value as normal.

#### 3.2.4. Evaluation Matrices

Many researchers and practitioners have optimised the effort estimation technique to assist the accuracy under various estimation standards. We implement the following standards to compare and evaluate the accuracy of the effort estimation model.

*(i) MRE*. One of the common criteria for evaluating the effort estimation process is the magnitude of relative error (MRE), which is computed using the following equation:

(9) $$\begin{array}{c} \text{MRE}=\left|\left(\frac{\text{Actual}-\text{Estimate}}{\text{Actual}}\right)\right|. \end{array}$$

*(ii) MMRE*. The MRE value is calculated from the dataset for each software item, while the mean magnitude of relative error (MMRE) calculates the average of N number of projects, as defined in the following equation:

(10) $$\begin{array}{c} \text{MMRE}=\frac{1}{N}\sum_{i=1}^{N}\text{MRE}.i. \end{array}$$

*(iii) MBRE*. MBRE is another measure that is commonly used to evaluate effort models. In recent years, it has been the average value of balanced relative error (MBRE) in software estimation research [12]. MBRE, in particular, is a useful evaluation standard because, as a balanced symmetric error measure, it penalizes both underestimation and overestimation at the same level and better handles the outline. MBRE is calculated in the following equation:

(11) $$\begin{array}{c} \text{MBRE}=\frac{1}{N}\sum_{i=1}^{N}\frac{\left|\text{Actual}-\text{Estimate}\right|}{\min\left(Estimte-Actual\right)}. \end{array}$$

*(iv) PRED*. The other most common metric is PRED(*I*), which represents all projects with an MRE percentage less than or equal to the I value. This standard, which is commonly used in the literature, is the proportion of projects completed with a given level of accuracy. In equation (12), pred(*x*) is defined.

(12) $$\begin{array}{c} \text{Pred}\left(x\right)=\frac{k}{N}, \end{array}$$

where *k* denotes the number of projects whose MRE is equal to or less than *x*, and *N* denotes the total number of projects. The most common value of *x* is 0.25, which is also used in this study. Pred(0.25) denotes the percentage of projects with MREs equal to or less than 25.

Estimation refers to the estimated value of the predicted efforts, actual refers to the actual workload or effort required to complete the project, and *N* denotes the number of projects.

#### 3.2.5. Experimental Result

In this section, optimization is carried out on all modes discussed above; it is performed on the NASA dataset, on semidetached mode, and on embedded projects, and the average of each mode is taken. The model also receives input from the COCOMO 81 dataset. During experiments, the KEMERER dataset is also used.

**Figure 4:**
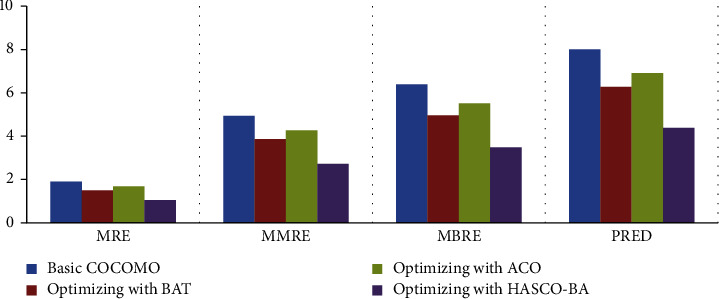
Comparison of NASA dataset.

*(i) Experiment 1*. Optimization is performed on the NASA dataset by using different nature-inspired algorithms. As results are shown in Figure 4, the MMRE value is decreased by using the meta-heuristic algorithms as compared to the basic COCOMO parametric model. All the datasets are divided into three folds, and then, the average is plotted on the graph, as shown in Figure 4. MMRE is decreased by using ACO, BAT, and HACO-BA algorithmic approaches. We noticed that the hybrid algorithmic approach HACO-BA shows a significant decrease as compared to other optimizing algorithms.

**Figure 5:**
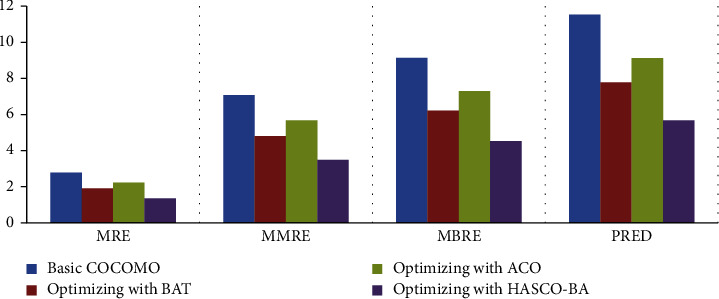
Comparison of COCOMO 81 dataset.

*(ii) Experiment 2*. The experimental results on the COCOMO 81 dataset reveal that the maximum value of MMRE is 7 as shown in Figure 5 as compared to the NASA dataset and the maximum value of MMRE is 5 as shown in Figure 5. Experimental results also show a decline in MMRE and other evaluation parameters while using the hybrid HACO-BA approach.

**Figure 6:**
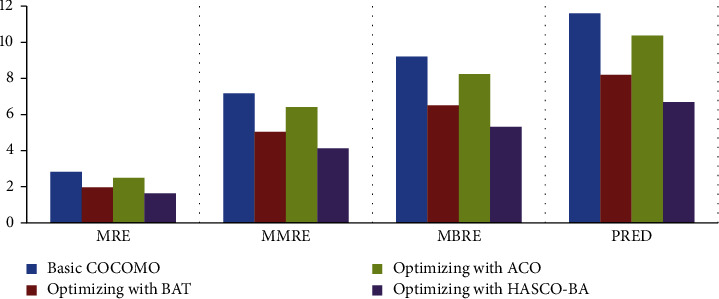
Comparison of KEMERER dataset.

*(iii) Experiment 3*. KEMERER dataset is also used to evaluate the performance of applied meta-heuristics approaches. In KEMERER dataset, we have 8 attributes, and to balance it with the above dataset, the rest of 7 attributes is assumed as normal whose value is equal to 1. Due to this, the decrease in MMRE is less as compared to NASA and COCOMO 81 datasets. On KEMERER, the dataset hybrid approach HACO-BA performs well compared to other meta-heuristic algorithms as shown in Figure 6.

In Table 4, valuation parameter values of the optimization models such as Basic COCOMO, BAT, ACO, and HACO-BA on different datasets are listed. These approaches are evaluated on MRE, MMRE, MBRE, PRED evaluation parameters using three different datasets; NASA, COCOMO, and KEMERER are listed.

## 4. Proposed Software Estimation Scheme Based on Hybrid Meta-Heuristic and Deep Learning Model

In the proposed system, we used the deep learning model. Deep learning is a type of artificial neural network architecture (ANN). ANN represents a significant early breakthrough in the field of artificial intelligence. The ANN model is exceptionally dynamic in solving complex problems in various machine learning application areas [11] in the real world, such as health, agriculture, finance, and automobile industry. At the moment, ANN in single, hybrid, or ensemble form is still an active research area [12], and its role in autonomous vehicles is expected to receive more attention in the future. ANN, on the other hand, is trained using backpropagation algorithms and has some limitations, such as falling into local minima and overfitting training data. As a result, many researchers advocate using nature-inspired algorithms to train ANNs to avoid challenges. For example, GA [24], ABC [34], CSA [36], and particle swarm optimization (PSO) [41] were used to train ANN and were found to be superior to the back propagation algorithm in terms of avoiding the local minima problem.

As stated above, deep learning is an ANN architecture with logical node weight updates and activation functions. Deep learning models and extracting high-level abstractions from large-scale data sets are useful when providing large-scale data [18]. Deep learning frameworks are based on cutting-edge machine learning research and are used to create new features for Silicon Valley startups.

Machine learning is not the same as traditional programming. In traditional programming, the program we write instructs the computer on how to complete the task. Aside from that, in machine learning, we do not tell the computer exactly what to do. Alternatively, we provide training data, and the machine learning algorithm uses this training data to develop its own rules for completing the task. Deep neural networks are used in a variety of applications.

- Image recognition, where you identify what objects are present in the images
- Image style transfer, where you can make a photograph look like, was painted in the style of a famous artist
- Language translation, where you translate from one human language to another
- Speech recognition, where you turn speech into text
- Business problem, to solve a typical business problem estimating sales

However, deep learning faces many limitations, but not restricted to the lack of system programs to achieve optimal or ideal parameter values, manual configuration of deep learning architectures, and lack of standard training methods and algorithms. Therefore, researchers have proposed many methods including nature-inspired algorithms to alleviate the challenges.

The application of nature-inspired calculations in profound learning is limited due to the need for cooperative energy between profound learning and nature-inspired calculations. As a result of the lack of synergy between deep learning and nature-inspired algorithms, the application of nature-inspired algorithms in deep learning is limited [19]. In the context of big data analysis, the author demonstrated the role of nature-inspired algorithms in deep learning. The study, however, argued that nature-inspired algorithms have a very limited application in deep learning methods [18].

**Figure 7:**
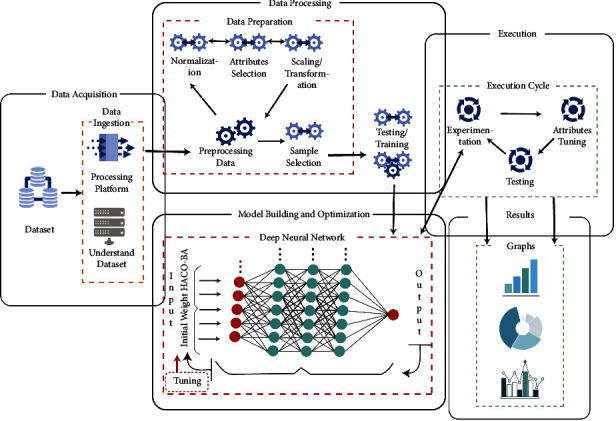
DNN model layers.

As the results are shown in Table 4, HACO-BA performs better among the other meta-heuristics algorithms. So, we applied the hybrid algorithmic approach to optimize the deep learning training process. HACO-BA is used to assign the best values of initial weights to the deep neural network. The proposed deep neural model is compared with [42, 46, 47] in terms of accuracy and time required for training. We have to find out the mean RE (MRE), mean magnitude of RE (MMRE), mean balanced residual error (MBRE), and percentage of prediction (PRED) and then compared it to the NN model. The block diagram of the proposed systems is shown in Figure7.

### 4.1. Data Acquisition and Processing

In data acquisition and for further processing, three different data sets are used in this approach. NASA dataset is used which has complete data for software cost estimation for 93 different software projects. These data are occupied by distinctive NASA centers for a long time. NASA dataset along with COCOMO 81 and KEMERER dataset have various attributes. For these attributes, different parameters are selected for effort and time estimation. From which, 15 common variables along with their descriptions are listed in Table 5 are taken, and these attributes are input to the deep neural network at the input layer. As this is supervised machine learning, we have to provide a value as the result value in the output layer, which is the total amount of effort needed to build the software product.

The important step is that we need to preprocess our data. In order to train the deep neural network, we want to scale all the numbers in each column of our dataset to be between the value of 0 and 1. This is because if the numbers in one column are large but the numbers in another column are small, the neural network training will not work very well. One of the best ways to do this is to use the MinMaxScaler object from the popular scikit-learn library. It is designed for exactly this purpose. In this method, first, we have to create a new MinMaxScaler, and then, we just need to pass in a feature range parameter, which tells it that we want all numbers scaled between 0 and 1.

### 4.2. Model Building and Optimization

In this section, model building and model optimization are presented.

#### 4.2.1. Model Building

This step involves creating the model in TensorFlow, which is used to create and deploy supervised machine learning models. Supervised machine learning is a type of machine learning in which the model is trained by providing the data as input and the expected result for that data. It determines how to convert the input into the output. When developing and deploying a supervised machine learning model, we always adhere to a process known as the train, test, and evaluate flow.

First, we write the code for our machine learning algorithm. We will accomplish this by constructing a computational graph of operations, in which, to begin, we will define each layer of the neural network and connect them so that data flow from the first to the last layer.

Then, we will add the placeholder node, which represents the data that will be fed into the neural network as input. Another placeholder node represents the neural network's output or the values predicted by the neural network. DNN has a total of five layers. There will be one input, one output, and three hidden layers between the neural network that will train to find the relationship between the inputs and the outputs. There are many different types of layers that can be used in a neural network, but we will stick with the most basic, a fully or completely connected neural network layer. That is, each node in each layer is linked to each node in the next layer.

Between layers, the first layer has 50 nodes, the second layer has 100 nodes, and the third layer has 50 nodes once more. Neurons are another name for nodes. Before training the model, the epoch is a hyperparameter to interpret in deep learning. When the entire dataset is passed forward and backward through the neural network only once, this is referred to as an epoch. We must set the training epochs to 100.

An epoch is another name for one full training pass over the training dataset. 50 epochs mean that we will do 50 iterations in our training loop to train our neural network, and similarly, 100 epochs mean 100 iterations.

Next, we need a way to measure the accuracy of the neural network's predictions. We will define the function that measures the each prediction accuracy during the training process. This is called a loss function. The loss function gets added to the graphs in its own operation. Then, we have to create an optimizer function that tells how we want to train the model.

When we run this function, it will perform one training step on our model. We will call this node the training operation. Bias is also an important parameter, and it means how far our forecast is from the actual value. In general, parameter algorithms have high biases, which makes them faster to learn and easier to understand, but they are usually less flexible.

The last part of defining this layer is multiplying the weights by the inputs and calling an activation function. An activation function outputs the result of the layer. We want the bias values for each node to default to zero.

#### 4.2.2. Model Optimization

The deep neural network performance is optimized through training by examining the loss function results and balancing the weight of each neural network layer to produce better results by increasing the number of epochs. With deep neural networks, a lot of research have gone into the best initial values to use for weights. Weight is a very important part of the deep neural network if a set of given weights is not correct, it will take time to train the network and will not make correct predictions, so we have optimised it using an meta-heuristic algorithm. For this purpose, we have used grey wolf optimization (HACO-BA) algorithms which are a nature-inspired algorithm to give the best values for initial weights to train the network more preciously. We access a model's layer by using model.layers. Here, we set a layer's weights with layer.setWeights() to obtain from grey wolf algorithmic optimizer. We have used code, like the following to set the optimised weights of each single layer: model.layers [1].getWeight().setWeights(.da.). Furthermore, we cannot set individual weights.

The variation in weights is decided by the learning rate. The learning rate is a parameter that apprises the optimizer on how far to move the weights in the direction of the gradient. We have adjusted the learning rate of our model. By using HACO-BA for weight initializers, the proposed deep neural network model produces better results in less time as in contrast to the neural network. Results of the proposed procedure are shown is compared with wavelet neural network-firefly algorithm morlet activation function (WNN-FA-MORLET) [42], deep modified neural network (Deep-MNN) [46], and evolutionary cost-sensitive: deep belief network (ECS-DBN) [47] is also listed in Table 6.

### 4.3. Performance Analysis and Evaluation of Deep Learning Model

In this section, performance analysis and evaluation of the deep learning model is discussed.

#### 4.3.1. Performance Analysis

The proposed deep neural network is compared with wavelet neural network-firefly algorithm morlet activation function (WNN-FA-MORLET) [42], deep modified neural network (Deep-MNN) [46], and evolutionary cost-sensitive deep belief network (ECS-DBN) [47] in terms of execution time required to train a model. Also, the proposed DNN is compared with the neural network in terms of accuracy achieved. Different software estimation datasets are given as input to the proposed DNN and find out whether the results change with a change in the dataset. In last, the proposed DNN is evaluated in terms of optimizing the evaluation matrices which is already defined in the subsection of Section 3 of this paper.

#### 4.3.2. Experimental Setup

The model is built using the TensorFlow. TensorFlow is an open-source software or a library for building and deploying machine learning methods. The *Python* programming language and other different libraries are also used to build the models.

#### 4.3.3. Dataset

As specified in the subsection of comparison of the existing meta-heuristic algorithms used for effort and cost estimation, three different most widely used dataset by the research community is used along with the China dataset which has a large number of software project data having 18 attributes in this experiments.

#### 4.3.4. Evaluation Matrices

The difference between the start and end time of process execution of models is calculated which is the total time required to test and train the model. Also in order to measure the cost/effort, we will calculate the mean square error between what the neural network predicts and what we expect it to calculate. To do that, we will call the tf.squared difference function and pass in the actual prediction and the expected value. Also, our expected value is *Y*.

Cost functions were included for a neural network, and the goal is to reduce the cost function. For this streamlined optimization problem, we use the GWO algorithm and variants of gradient descent where the model parameters (here weights and biases in the network) are rationalized in a way to reduce the cost function. All the data sets are used one by one for the training phase and for the testing phase. As this is a computational graph, there is no single start or end. We can start processing at any node in the graph, before we can perform any of the operations in our graph, we have to generate a session. Once the session object is created, we can ask it to run any operation in the graph. To train the model, we will call the training operation over and over. Each time the training operation runs, we will pass a new training data that will be used for that training pass. And then, we will check the current accuracy by calling the loss function. While the training process is running, we can watch the results graphically using a separate tool called Tensor Board. Different evaluation matrices which are specified in the sun section of Section 3 are also evaluated.

### 4.4. Experimental Result

Various experiments are carried out, and their results are compared in terms of time required for training, accuracy, and various evaluation matrices.

#### 4.4.1. Experimental 1

**Figure 8:**
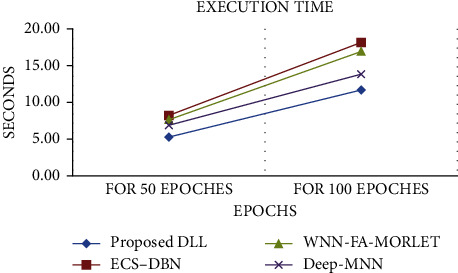
Comparison with literature.

In this experiment, the proposed scheme is differentiated with several DNN and NN models that include a deep modified neural network (Deep-MNN) [46], evolutionary cost-sensitive deep belief network (ECS-DBN) [47], and wavelet neural network-firefly algorithm morlet activation function (WNN-FA-MORLET) [42] in term of time required for training. With 50 epochs and with 100 epochs, we run the training and testing process, and the results reveal that the HACO-BA-DNN uses the less execution time appears in Figure 8 as compared to other nature-inspired algorithms.

#### 4.4.2. Experimental 2

**Figure 9:**
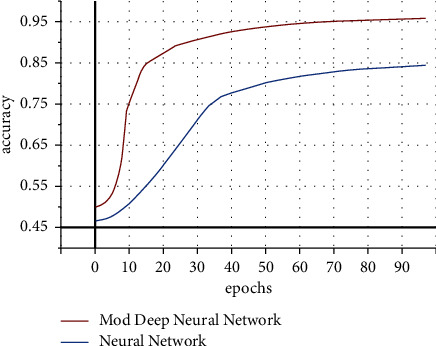
Comparison between NN and proposed DNN.

In this experiment, the proposed DNN is evaluated with neural network in terms of achieved accuracy. The process is executed upto 100 epochs, and the accuracy is accomplished by the proposed DNN approach is almost 98%. While NN achieved accuracy upto 85% on the same datasets as shown in Figure 9, the HACO-BA-DNN performs better in terms of accuracy as compared to NN.

#### 4.4.3. Experimental 3

**Figure 10:**
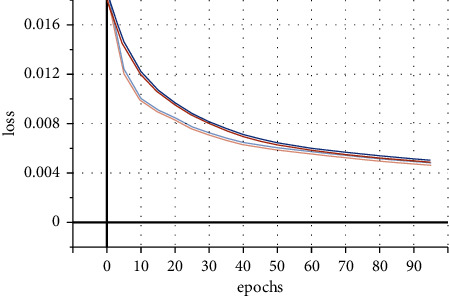
Using different datasets.

Various datasets of software estimation are given as input to the HACO-BA-DNN to find the change in results by changing the dataset. For this purpose, we define the function which is known as the loss function that measures the accuracy of each prediction during the training process.  Figure 10 demonstrates that there is no visible change seen in results when we change the dataset from NASA to COCOMO, KEMERER, or China.

#### 4.4.4. Experimental 4

**Figure 11:**
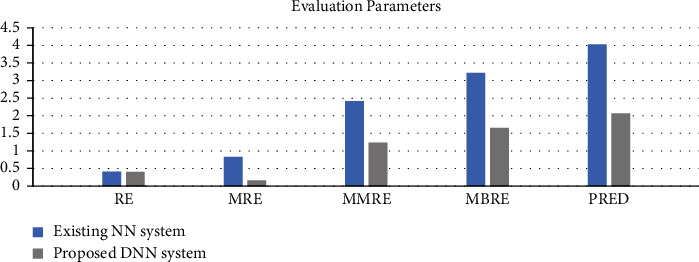
Comparison of NN with proposed DNN.

The HACO-BA-DNN is evaluated as compared to NN by using various performance evaluation matrices, which are already defined in the subsection of comparison of the existing meta-heuristic algorithms. The results in Figure 11 show that the proposed DNN performs better in terms of reduction in MRE, MMRE, MBRE, and PRED. The smaller the value of the performance matrices shows that more results improve, and better software cost and effort estimation is achieved.

## 5. Discussions

### 5.1. Answers to Research Questions

RQ1, which of the meta-heuristic algorithm, has the lowest MRE, MMRE, MBRE, and PRED.

ACO and BAT along with their hybrid meta-heuristic algorithm, that is, HACO-BA has been implemented and their performance, is tested in terms of reduction in MRE, MMRE, MBRE, and PRED. As results show that all the algorithms reduced evaluation parameters as compared to the basic COCOMO parametric model, HACO-BA performs better among all other algorithms.

RQ2: whether the performance of the meta-heuristic algorithm changes/varies by changing the dataset.

All the meta-heuristic algorithms have been implemented, and their performance is tested and evaluated on three different publicly available data sets (NASA, COCOMO, and KEMERER), and we take MMRE as an evaluation parameter to check the performance of all three datasets. The results shows that the performance of HACO-BA is better as compared to the BAT, ACO, and Basic COCOMO as shown in Table 4.

RQ3: nature-inspired and meta-heuristic algorithms combined with deep learning can improve the software estimation process.

The proposed DLL takes less execution time as compared to other algorithms taken from the literature review. So it improves the software estimation process as shown in Figure 9.

RQ4: whether the performance of meta-heuristic-based deep learning algorithm changes by changing the dataset.

By changing the dataset, the performance of the proposed meta-heuristic deep learning architecture does not change. As shown in Figure 10, the two lines show that, with the passage of execution, both the data sets achieve almost the same accuracy.

RQ5: whether meta-heuristic-based deep learning algorithm performs better than the NN-based approach for software estimation.

Blue line shows the existing neural network approach.

Orange line shows the proposed deep neural network.

As shown in Figure 11, our proposed deep neural network performs better in terms of accuracy. NN takes more time/epochs as compared to HACO-BA-DNN to train its network to achieve better results in terms of software development effort reduction.

## 6. Conclusions

The proposed method investigates the efficacy of estimating efforts by combining ACO, BAT, and HACO-BA with COCOMO for effort estimation using optimised coefficients. To test the effectiveness of the proposed method, three datasets are used: Nasa, COCOMO 81, and KEMMER. MMRE values are improved in each optimised scenario, with HACO-BA outperforming all others. A new method that combines meta-heuristics and DNN is also introduced. As a result of the results, it was determined that the optimised method produces better estimates than the basic method in terms of effort and cost estimation in all models. The experimental results show that the hybrid HACO-BA performs better for tuning COCOMO II than ACO and BA and that HACO-BA performs better in DNN optimization in terms of execution time and accuracy than NN.

## 7. Future Work

In the future, we will improve the estimation models by experimenting with new methods and incorporating cloud computing for estimating purposes in order to obtain more comprehensive results in the future. Researchers and practitioners in [59] and [61] used the Strawberry Plant heuristics approach for software cost estimation and for energy management. In [60, 62], Grey Wolf and Bacterial Foraging approaches are used in smart grids for energy management and heterogeneous generalized signcryption to maintain the data integrity for estimation.

## Figures and Tables

**Algorithm 1:**
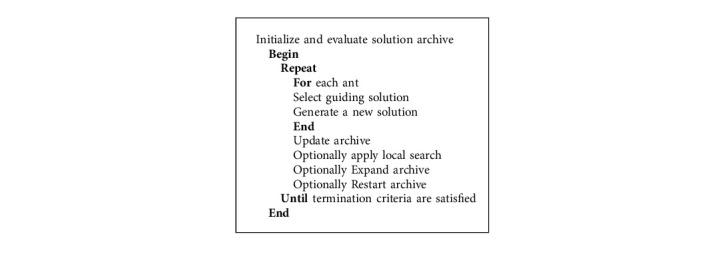
Ant colony optimization (ACO).

**Algorithm 2:**
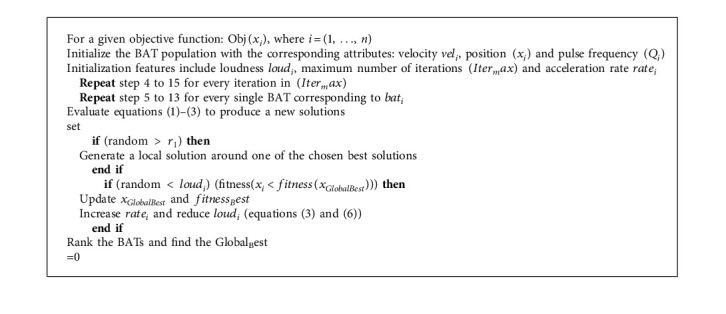
BAT algorithm (BA).

**Algorithm 3:**
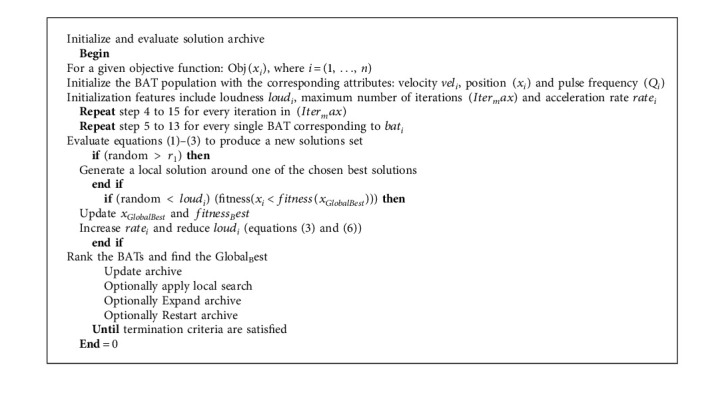
Hybrid ant colony and BAT algorithm (HACO-BA).

**Table 1:** Notation guide.

Notations	Abbreviation
ML	Machine learning
ACO	Ant colony optimization
BA	BAT algorithm
MMRE	Mean magnitude relative error
NN	Neural network
DL	Deep learning
COCOMO	COnstructive COst Mode
BCO	Bee colony optimization
PSO	Partial swarm optimization (PSO)
COCOMO	COnstructive COst Model
SLOC	Source lines of code
RQ	Research questions
MAR	Mean absolute residual
RBFNN	Radial basis function NN
RF	Random forest
COA	Chaos optimization algorithm
ABC	Artificial bee colony
OPSO	Optimized particle swarm optimization
MBRE	Mean balanced relative error
INGPS	IdeNtitybased generalised proxy signcryption

**Table 2:** Estimation method and limitations.

Estimation method	Limitations

Estimation by analogy	Subjective selection of correlation standards and dispute identification process (confidence level)
	Requires analogous project for comparison from historical data from database
	These analogous projects are rarely available in software development

Decomposition and bottom-up (WBS-based)	It may be time-consuming for large or even medium-sized projects
	High risk of ignoring system-related tasks such as testing, integration, and configuration is high
	This method may lead to underestimation due to lack of project information at early stage

Parametric models (SLIM, SEERSEM)	Usually does not take into account the project team's skill set specific to the organization's software and project management culture
	Modern methods of code reuse, code less programming, and various agile development methods for software development may not be feasible
	Highly dependent on programming language

Expert estimation (Delphi, PERT, planning poker)	These methods rely on the experience, knowledge, and perception of experts, and there may be deviations or biased, which often lead to overestimation or underestimation
	All the factors used by experts in the estimation process are unable to justify and quantify

Size-based estimation models (use case, FPA, sTory points)	Requires trained personnel which is not easily available
	High effort and cost is required for the application of large projects
	Due to limited information, using this method in the early stages of a project may result in inaccurate estimates

**Table 3:** Comparison between existing approaches.

Refer ence	DL/ML/ANN	Meta-heuristic algorithm	Dataset	Evaluation parameter	Contributions
[42]	NN	Fiery algorithm, BAT algorithm	COCOMO81, NASA, MAXWELL, China	MRE, MMRE, pred, MDMRE	Hybrid model for effort estimation
[45]	ANN	Firefly	COCOMO81, NASA, MAXWELL, China	MMRE, MdMRE, PRED	Hybrid model for cost estimation
[46]	DNN	Cuckoo, hybrid PSO	COCOMO	RE, MRE, MMRE, MARE, PRED, execution time	Hybrid model for cost estimation
[11]	DNN	GA	KC1, KC2, CM1, PC1, JM1	Accuracy, precision, F-score, recall, sensitivity	Defect prediction
[47]	DL	Evolutionary algorithm	KEEL dataset repository	Accuracy, G-mean, precision, F-score, computational time	Hybrid of DBN and ADE for imbalanced classification
[48]	NN	GA, PSO	N/A	Survey	Possibility to apply on DL
[49]	NN	Cuckoo	COCOMO	MMRE, standard deviation	Improve cocomo
[50]	ANN	Cuckoo	COCOMO81, NASA	MMRE, PRED, computational time	Hybrid model
[51]	GWO, HSA	SBA	NASA	MRE, MMRE	Hybrid model

**Table 4:** Parameter evaluation using different datasets.

Optimization models	NASA				COCOMO				KEMERER			
	MRE	MMRE	MBRE	PRED	MRE	MMRE	MBRE	PRED	MRE	MMRE	MBRE	PRED
Basic COCOMO	1.93	4.95	6.39	8.04	2.76	7.07	9.12	11.49	2.79	7.15	9.23	11.62
BAT	1.51	3.87	5.00	6.30	1.87	4.78	6.16	7.77	1.97	5.04	6.51	8.20
ACO	1.67	4.28	5.53	6.96	2.19	5.61	7.24	9.12	2.49	6.38	8.23	10.37
HACO-BA	1.06	2.72	3.51	4.42	1.35.	3.47	4.47	5.63	1.61	4.12	5.31	6.69

**Table 5:** Selected parameters for effort and time estimation.

Variables	Description	Type	Role
Analyst's capability	Ability to learn and examine the system	Nominal	Input
Application experience	Basic application knowledge and skills	Nominal	Input
Process complexity	Event and tasks assessment that make the process	Nominal	Input
Database size	Large and complicated database	Nominal	Input
Modern programming practice	Updated method used for development	Nominal	Input
Programmer's capability	Knowledge and skill of programmer	Nominal	Input
Required software reliability	Failure-free probability of software	Nominal	Input
Schedule constraint	Earlier identify limitations on project schedule	Nominal	Input
Main memory constraint	Memory needs to effectively and efficiently completes several operations	Nominal	Input
Time constrain for CPU	Processing time to complete an action	Nominal	Input
Turnaround time	Amount of time required to complete a specific process	Nominal	Input
Virtual machine experience	Need for experience to operate on virtual systems	Nominal	Input
Use of software tools	Used of various modern framework	Flag	Input
Machine volatility	Experience and valuable knowledge to operate several machines	Nominal	Input
Effort	Efforts or resources required for development	Continuous	Output

**Table 6:** Evaluation of execution time.

Methods	50 Epochs	100 Epochs
WNN-FA-MORLET	7.68	16.91
Deep-MNN	6.96	13.84
ECS-DBN	8.29	18.23
HACO-BA-DLL	5.32	11.7

## Data Availability

The data used to support the findings of this study can be obtained from the corresponding author.
